# Predictors of poor outcome in the acute phase after a first-ever stroke in a population-based study in Matão, Brazil

**DOI:** 10.1055/s-0045-1804924

**Published:** 2025-03-19

**Authors:** Cesar Minelli, Esther Maria Langhi Chiozzini, Liliana Tiene Ujikawa, Geraldo Cassio dos Reis, Millene Rodrigues Camilo, Octavio Marques Pontes-Neto

**Affiliations:** 1 Universidade de São Paulo, Faculdade de Medicina de Ribeirão Preto, Departamento de Neurociências e Ciências do Comportamento, Ribeirão Preto SP, Brazil.; 2 Hospital Carlos Fernando Malzoni, Departamento de Neurologia, Matão SP, Brazil.

**Keywords:** Stroke, Prognosis, Risk, Population, Prospective Studies

## Abstract

**Background** The data on predictors of poor outcomes for stroke patients in middle-income countries are lacking.

**Objective** To identify in the acute phase after a first-ever stroke (FES) predictors of a poor outcome within 3 months and 1 year in a population-based study in the town of Matão, state of São Paulo, Brazil.

**Methods** We prospectively investigated the characteristics of patients with FES in Matão, from a prospective study, in a population-based stroke registry, from 2015 to 2020. Poor outcome was defined as a modified Rankin scale (mRS) score of 3 to 6, assessed at 3 months and 1 year of follow-up. The association between predictors and poor outcome was analyzed by logistic regression analysis.

**Results** Of the 783 patients, the final sample consisted of 378 subjects for analysis. The mean age was 69.2(± 14.3) years, and 43.1% of patients were female. At 3 months and 1 year after a FES, 50.4% and 47.1% of stroke patients were classified as poor prognosis, respectively. Older age, female gender, hemiplegia, aphasia, subarachnoid hemorrhage, and comorbidities present in the acute phase were the predictors associated with a poor outcome.

**Conclusion** Non-modified and potentially modified predictors increase the risk of a poor FES prognosis in a population-based study from a middle-income country. Interventions focusing on these target populations and improving access to prevention and stroke management in the acute phase are necessary.

## INTRODUCTION

Identifying predictors of poor outcomes in stroke patients upon admission is crucial for indicating appropriate therapy, planning rehabilitation, estimating costs, and meeting patient and family expectations. However, most data on stroke outcome predictors come from studies conducted in high-income countries 1 2 3 , while data on stroke outcome predictors from population-based studies in middle income-countries are scarce. 4 5

The purpose of the present study was to identify admission predictors of poor outcome at 3 months and 1 year after a first-ever stroke (FES) in a population-based study in the town of Matão, state of São Paulo, Brazil.

## METHODS

### Study design

The Matão Preventing Stroke (MAPS) is a prospective study, community-based, in the town of Matão, and registered in Clinical Trials as NCT03391102. We collected data from August 1, 2015, to July 31, 2020.

### Site of investigation

The site of investigation and case ascertainment has been previously described. 6 Briefly, Matão is a town in the state of São Paulo, with a territorial area of 524,899 km ^2^ , and it is located 300 km northwest of the state capital. 6 Matão's population was of 80,327 inhabitants in 2022. The monthly basic salary is US 236.20. Basic sanitation and electricity are present in 95% of the town. 7 The Estratégia de Saúde da Família (Family Health Strategy [FHS]) assists almost of the entire population of Matão. Family Health Strategy is a community-based approach to providing primary health care. 8

The whole population of Matão and of two other small towns is served by the only hospital in the town, Hospital Carlos Fernando Malzoni, which provides local health services to public and private patients. This general hospital has 198 beds, including 25 intensive care unit (ICU) beds. At the time when the study was carried out, the only computed tomography (CT) and magnetic resonance imaging (MRI) units were located in this hospital. Their emergency room (ER) is the only department in the town, providing care for 5,500 to 6,800 cases per month. The management of acute stroke patients includes hospitalization and a brain CT scan performed as soon as possible. The hospital is reference to the only private emergency care facilities and the only nursing home for suspected stroke patients.

Patients were assessed 24 h after the stroke symptoms. Suspected stroke patients were referred to the ER of Hospital Carlos Fernando Malzoni by general physicians, nurses, and community health agents. Admission, discharge charts, and daily checks of emergency consultation were performed (hot pursuit). Neurologists who do not work at the hospital, and all physicians not neurologists were instructed to refer suspected acute stroke patients to the research team. All brain CT and MRI of patients with suspected stroke were checked. Monthly check-ins were undertaken of nursing homes and outpatient clinics. All death certificates were checked monthly to search for suspected stroke patients (cold pursuit).

### Research team

The research team consisted of two neurologists (CM and LTU), one nurse coordinator (EMLC), and five nurses. At least one neurologist from the research team assessed suspect stroke patients.

### Inclusion criteria

The inclusion criteria were patients living in Matão, regardless of age. Every stroke case was registered; however, only FES cases were included in the analysis.

### Exclusion criteria

Patients were excluded when there was a clinical evidence of a previous stroke event. Patients from other cities, who did not fulfill the clinical criteria for stroke, and with brain imaging findings incompatible with a clinical suspicion of stroke were also excluded.

### Assessment, classifications, definitions, and follow-up

A diagnosis of clinical stroke was defined as the acute onset of neurological deficits lasting more than 24 hours, with no apparent cause other than a cerebrovascular accident. 9 Stroke diagnosis was confirmed by clinical examination and by CT scan or MRI. Subjects were assessed with the National Institutes of Health Stroke Scales (NIHSS) validated for the Brazilian population. 10 National Institutes of Health Stroke Scales scores from 0 to 3 were considered to indicate a mild stroke, scores of 4 to 9 to indicate a moderate stroke, and scores of 10 or higher to indicate severe strokes.

Predictors of poor prognosis were classified as demographics, socioeconomic condition, comorbidities, vital signs, and neurological status at admission. Also, patients were classified in terms of type of stroke and subtype of ischemic stroke according to the Oxfordshire Community Stroke Project (OCSP) classification, 11 that is, lacunar infarct (LACI), partial anterior circulation infarct (PACI), total anterior circulation infarct (TACI), and posterior circulation infarction (POCI). The demographic variables were age and gender. Socioeconomic predictors were defined as follows: access or not to FHS care; number of years of education, dichotomized into 0 to 4 and ≥ 5 years; marital status, classified as married, divorced, single, or widow(ed); work status, classified as retired, pensioner, or active worker; basic monthly salary dichotomized into < 2 and ≥ 2 monthly minimum wages; family status (if patient had sons and/or daughters); and time between the onset of stroke and hospital arrival. Time between stroke onset symptoms and arrival at the hospital were categorized into intervals of less than 3 hours, 3 to 4.5 hours, or more than 4.5 hours. Comorbidities were defined as follows: arterial hypertension, diabetes mellitus, and/or dyslipidemia were diagnosed if the patient had a history of these comorbidities or was under specific treatment. 6 A history of angina or myocardial infarction defined coronary disease. If a patient had a history of intermittent claudication and peripheral disease, peripheral artery disease was diagnosed. Valve disease was diagnosed as a self-reported history of this condition. Electrocardiogram findings defined atrial fibrillation. Heart failure was diagnosed as a self-reported history of heart failure or if the patient was under treatment for heart failure. Chagas Disease was diagnosed if the patient had a previous diagnosis of this disease. Preexisting dementia was diagnosed according to family information or to the use of medical treatment for dementia. Previous transient ischemic attack (TIA) was defined as a history of acute onset of neurological deficits lasting less than 24 hours, with no apparent cause other than a cerebrovascular accident. Smoking status was defined as nonsmoker or current smoker, that is, individuals who had smoked any tobacco products in the last 12 months. Alcohol use was defined as recent alcohol use and consumption of at least 30 g of ethanol for men and 15 g of ethanol for women at least 3 times a week. Vital signs at admission were heart rate, systolic and diastolic blood pressure, and axillary temperature. Neurological status assessed at admission was defined as presence of hemiplegia, hemiparesis, hemi-hypoesthesia, visual fields, conjugated eye deviation, somnolence or coma, dysphagia, aphasia, and visual or sensorial neglect. Ischemic stroke (IS) was defined as an episode of sudden onset of neurological symptoms and findings of brain ischemia on neuroimaging. Intracerebral hemorrhage (IH) was defined when sudden neurological finds are associated with bleeding in intraparenquimal brain tissue finding on CT scan, while subarachnoid hemorrhage (SAH) was classified based on clinical, CT scan and cerebrospinal fluid findings. 12 Undetermined stroke was defined as a stroke that could not be classified as any of the previous types since a CT scan or autopsy was not performed. 12

The dependent variable was outcome after stroke, assessed by the Modified Rankin Scale (mRS). 10 The scale runs from 0 (no symptoms) to 6, (death). Poor outcome was defined as an mRS score of 3 to 6. Patients were assessed by phone at 3 months and 1 year of follow-up.

### Data analysis

Categorical variables were analyzed by Fisher's exact test and assessed as count and percentage. Continuous variables were analyzed with the Student's t-test and assessed as mean or median with standard deviation (SD) or interquartile range, depending on data distribution. A multicollinearity test was performed, and multiple imputation was used to complete the presence of missing data. 13 First, the univariable model was used to identify the possible independent risk factors for mortality. The clinically significant variables and all variables with a *p* -value less than 0.1 were included in the multivariable analysis model. Second, multivariable analysis and the step-backward method were used to identify the independent prognostic factors for a poor prognosis. The odds ratios (OR) and their 95% confidence intervals were calculated from this logistic regression model. The level of significance of 5% was considered after Bonferroni adjustment (0.05/number of tests performed). Statistical analysis was performed with IBM SPSS Statistics for Windows (IBM Corp., Armonk, NY, USA) software, version 22.0.

### Ethics

Written informed consent was provided by all patients. If the patient was incapacitated, the informed consent was provided by their relatives. The study was evaluated and approved by the Institutional Ethics Committee of Centro Universitário Barão de Mauá (number 296.915/2013).

## RESULTS

A total of 783 patients with suspected stroke were studied during the 2015 to 2020 period. After excluding subjects with a previous stroke (20.1%), from other cities (12.6%), with stroke mimics (10.6%), with TIA (7%), and cerebral venous thrombosis (0.63%), those lost to follow-up (0.63%), and 0.13% with unclassified types of stroke , our final sample consisted of 378 patients. At 3 months and 1 year after a FES, 50.4% and 47.1% of stroke subjects were classified as having a poor prognosis, respectively. All patients were subjected to a CT scan within the first 24 h. The mean age was 69.2(± 14.3) years, and 43.1% of the patients were female. The mean years of schooling was 4.5 (± 3.9), and 61.1% of the patients were retired. Regarding marital status, 57.9% were married, 25.1% were widowed, 9.8% were divorced, and 7.1% were single; 89.4% had children, and 77.7% had an income of less than 2 monthly minimum wages. The time between stroke onset symptoms and arrival at the hospital was less than 3 hours for 56.1% patients, 3 to 4.5 hours for 11.4%, and more than 4.5 hours for 32.5%. Hypertension (76.1%), diabetes (34.1%), and smoking (23.0%) were the most frequent comorbidities. A family history of stroke was reported by 21.1%. The median NIHSS score was 5 (interquartile range [IQR]: 3–15), with stroke being scored as mild in 34.8% of patients, moderate in 30.2%, and severe in 35.0%. Hemiparesis (52.6%), aphasia (45.0%), conjugated eye deviation (18.5%), and somnolence/coma (18.9%) were the most frequent neurological findings upon examination. The mean systolic and diastolic blood pressure, heart rate, and axillary temperature were 160.0(± 35.1) mmHg, 91.0(± 19.8) mmHg, 81.0(± 17.5) beats/min, and 36.1 ^o^ C, respectively. Ischemic, hemorrhagic, and SAH were found in 79.4%, 13.8%, and 6.9% of stroke patients, respectively. Regarding the subtypes of ischemic stroke, 51.5% were classified as LACI, 19.9% as PACI, 22.5% as TACI, and 6.1% as POCI.

In unadjusted logistic regression, female gender, older age, fewer years of education, retirement, widowhood, atrial fibrillation, dementia, a past history of stroke in the family, hemiplegia, conjugate eye deviation, somnolence/coma, aphasia, neglect, hemorrhagic stroke and SAH, and TACI according to OCSP were associated with a poor prognosis at 3 months and 1 year after hospital admission due to FES ( Table 1 ). Having children, being an active worker, having 5 or more years of education, and earning 2 or more monthly minimum wages were associated with better prognosis.

**Table 1:** Univariable logistic regression analysis of demographics, socio-economic, comorbidities and clinical status for outcome at 3 months and 1 year after a FES

Modified Rakin Scale		3 months			1 year		
		0–2	3–6	*p*	0–2	3–6	*p*
Overall: n (%)		188 (49.6)	190(50.4)	0.87	200 (52.9)	178 (47.1)	0.26
Gender: n (%)	Male	128 (68.1)	87 (45.8)	< 0.001	136 (68.0)	79 (44.4)	< 0.001
	Female	60 (31.9)	103 (54.2)		64 (32.0)	99 (55.6)	
Age (years): mean(± SD)		65.3(± 13.1)	72.9(± 14.6)	< 0.001	64.9(± 13.3)	73.9(± 13.9)	< 0.001
Years of education (%)	0–4	130 (47.1)	146 (52.9)	0.01	139 (50.2)	138 (49.8)	0.01
	≥ 5	56 (62.9)	33 (37.1)		60 (67.4)	29 (32.6)	
Retired: n (%)		116 (50.2)	115 (49.8)	0.91	121 (52.4)	110 (47.6)	0.61
Pensioner: n (%)		10 (23.3)	33 (76.7)	0.001	11 (25.6)	32 (74.4)	< 0.001
Working before stroke		77 (70.6)	32 (29.4)	< 0.001	85 (78.0)	24 (22.0)	< 0.001
Marital status: n (%)	Married	131 (59.8)	88 (40.2)	< 0.001	140 (63.9)	79 (36.1)	< 0.001
	Divorced	17 (45.9)	20 (54.1)		18 (48.6)	19 (51.4)	
	Single	13 (48.1)	14 (51.9)		13 (48.1)	14 (51.9)	
	Widow(ed)	28 (29.5)	67 (70.5)		31 (32.6)	64 (67.4)	
Sons/daughters		177 (52.4)	161 (47.6)	0.007	188 (55.6)	150 (44.4)	0.013
Monthly minimum wages: n (%)	< 2	132 (46.2)	154 (53.8)	0.003	142 (49.7)	144 (50.3)	0.001
	≥ 2	57 (62.0)	35 (38.0)		60 (65.2)	32 (34.8)	
Time onset symptoms/hospital arrival	< 3:00 h	100 (47.2)	112 (52.8)	0.35	75 (61.0)	48 (39.0)	0.11
	3–4:30 h	21 (48.8)	22 (51.2)		104 (49.1)	108 (50.9)	
	> 4:30 h	68 (55.3)	55 (44.7)		23 (53.5)	20 (46.5)	
Comorbidities: n (%)	Alcohol abuse	39 (58.2)	12 (17.9)	0.30	39 (57.4)	29 (42.6)	0.48
	Smoking	41 (47.7)	46 (52.0)	0.54	43 (49.4)	44 (50.6)	0.39
	Atrial fibrillation	5 (29.4)	12 (70.6)	0.01	5 (24.0)	12 (76.0)	0.002
	Heart failure	4 (28.6)	10 (71.4)	0.09	4 (28.6)	10 (71.4)	0.06
	Preexisting dementia	1 (9.1)	10 (90.9)	0.01	1 (9.1)	10 (90.9)	0.003
History of stroke in family		51 (63.8)	29 (36.9)	0.01	58 (72.5)	22 (27.5)	< 0.001
Vital signs at admission: meran(± SD)	Mean SBP (mmHg)	157.5(± 30.1)	163.42(± 39.9)	0.10	157.1(± 30.2)	164(± 40.3)	0.05
	Mean DBP (mmHg)	89.9(± 16.9)	92.2(± 22.9)	0.27	89.8(± 16.9	92.5(± 23.3)	0.20
	Heart rate (beats/min)	79.1(± 15.9)	82.2(± 19.7)	0.10	78.7(± 15.8)	82.9(± 19.9)	0.03
	Mean axillary temperature in °C	36.1(± 0.41)	36.1(± 0.54)	0.59	36.2(± 0.40)	36.1(± 0.57)	0.40
Neurological status at admission: n (%)	Hemiplegia	9 (18.4)	40 (81.6)	< 0.001	11 (22.9)	37 (77.1)	< 0.001
	Hemiparesis	100 (52.6)	91 (47.6)	0.40	111 (57.8)	81 (42.2)	0.76
	Hemi-hypoesthesia	12 (75)	1 (7.7)	0.02	11 (68.8)	5 (31.3)	0.02
	Visual fields	8 (57.1)	6 (42.9)	0.60	8 (57.1)	6 (42.9)	0.79
	Conjugate eye deviation	6(5.95)	64 (94.05)	< 0.001	7 (10.3)	61 (89.7)	< 0.001
	Somnolence/coma	16 (27.1)	43 (72.9)	< 0.001	17 (28.8)	42 (71.2)	< 0.001
	Dysphagia	9 (42.9)	12 (57.1)	0.65	9 (42.9)	12 (57.1)	0.37
	Aphasia (severe or mute)	11 (15.4)	81 (84.6)	< 0.001	13 (17.7)	79 (82.3)	< 0.001
	Neglect (visual or sensorial)	9 (19.1)	38 (80.9)	< 0.001	10 (21.3)	37 (78.7)	< 0.001
Stroke classification: n (%)	Ischemic	160 (53.3)	140 (46.7)	0.04	172 (57.3)	128 (42.7)	0.01
	Hemorrhagic	20 (38.5)	32 (61.5)		21 (40.4)	31 (59.6)	
	SAH	9 (34.6)	17 (65.4)		9 (34.6)	17 (65.4)	
Oxfordshire classification: n (%)	LACI	86 (72.3)	33 (27.7)	< 0.001	90 (75.6)	29 (24.4)	< 0.001
	PACI	21 (45.7)	25 (54.3)		22 (47.8)	24 (52.2)	
	TACI	5 (9.6)	47 (90.4)		6 (11.5)	46 (88.5)	
	POCI	10 (71.4)	4 (28.6)		11 (78.6)	3 (21.4)	

Abbreviations: DBP, diastolic blood pressure; FES, first-ever stroke; LACI, lacunar infarction; PACI, partial anterior circulation infarction; POCI, posterior circulation infarction; SBP, systolic blood pressure; SAH, subarachnoid hemorrhage, TACI, total anterior circulation infarction.

After adjusting for potential confounders, multivariable logistic regression analyses showed that older age, female gender, hemiplegia, aphasia, and SAH were associated with a poor prognosis at 3 months ( Table 2 ). Being single and having children were protective factors at 3 months. Preexisting dementia and atrial fibrillation were nonsignificant tendencies towards a poor prognosis at 3 months. At 1 year after an FES, the predictors of poor outcome at admission were older age, widowhood, dementia, heart failure, hemiplegia, aphasia, and SAH ( Table 2 ). Atrial fibrillation presented a nonsignificant tendency to poor prognosis at 1 year. Working before the stroke and having children were protective factors 1 year after an FES.

**Table 2:** Multivariable binary logistic regression for poor prognosis at three months and one year after a FES

	3 months			1 year		
	OR	95%CI	*p*	OR	95%CI	*p*
Age ^†^	1.04	1.02–1.06	< 0.001	1.04	1.01-1.06	0.002
Female	1.87	1.13–3.08	0.02	----	---	----
Widow (ed)	----	----	---	2.15	1.15–4.04	0.02
Working before stroke	---	---	---	0.48*	0.24–0.95	0.035
Single	0.12	0.02–0.64	0.01	----	---	---
Sons/daughters **	0.06	0.01–0.27	< 0.001	0.2*	0.05–0.75	0.02
Preexisting dementia	8.15	0.98–67.91	0.05	10.09	1.16–87.55	0.04
Atrial fibrillation	2.81	1.00–7.91	0.05	2.73	0.94–7.93	0.06
Heart failure	---	----	---	4.64	1.19–17.99	0.03
Hemiplegia	3.19	1.63–6.26	0.001	2.72	1.44–5.70	0.003
Aphasia	1.80	1.11–2.92	0.02	2.22	0.02–087	0.002
SAH	3.67	1.38–9.76	0.01	6.62	2.38–18.41	< 0.001

Abbreviations: 95%CI, 95% confidence interval; FES, first-ever stroke; OR, odds ratio; SAH, subarachnoid hemorrhage.

Note: ^†^ Every year of increase in age increases the chance of having a poor prognosis by 4%.

## DISCUSSION

Our population-based study conducted in the town of Matão, Brazil, revealed that factors such as age, female gender, comorbidities, hemiplegia, aphasia, and SAH are predictors of poor outcome 3 months and 1 year after hospital admission due to an FES. Having siblings and working before the stroke were identified as protective factors against poor prognosis after an FES. These findings are consistent with previous population-based studies in high-income countries, which have shown that factors such as older age, low level of activity before the stroke, 14 comorbidities, recurrent stroke, depression, 15 stroke severity, severe hemiparesis, 16 and hemorrhagic stroke 17 were associated with poor outcome after a stroke. Taken together, these results reinforce the importance of early identification of stroke risk factors and prompt intervention to prevent stroke and improve outcomes, especially in middle- and low-income countries.

Few studies in Latin America and Brazil have assessed poor prognosis after an FES. In Ñandum, Chile, 4 (19) 61% of FES patients died or were disabled, and in Joinville, Brazil, 5 45% scored 3 to 6 on the Rankin scale 1 year after a stroke. However, none of these studies assessed risk predictors of poor prognosis. The Study of Stroke Mortality and Morbidity (The EMMA study), a community-based hospital study conducted in the São Paulo capital, Brazil, reported that the association of low educational level with diabetes was a prognostic factor associated with higher mortality in ischemic stroke (EMMA). 18 The same study group found that lack of formal education and a burden of comorbidities were independent risk factors. 19 Another hospital-based multi-center study conducted in Fortaleza, capital of the state of Ceará, reported that older age, prestroke disability, and a depression in the level of consciousness were independent factors of poor prognosis. 20 We found just one study in a low income country about outcome after an FES. 21 In Guinea, lower admission NIHSS score, brain CT imaging within 24 h of symptoms onset, and lower mean arterial blood pressure were positive predictors of a favorable stroke outcome.

The strength of our research is that, instead of being a hospital-based study, it is a population-based study with the advantage of capturing all FESs in a defined, whole town population. This methodology permits assessing the incidence of FES, avoiding selection bias in subjects exposed to the risk of stroke. 22 Therefore, outcomes of population-based studies are more representative of a community population than hospital-based studies. Another positive aspect is that all subjects were subjected to a CT scan within 24 hours, and just 1 patient had unclassified type of stroke. Also, only 0.63% of the subjects were lost to follow-up. However, our study also has some limitations, such as no information about previous disability before the FES index and just 1 year of follow-up, whereas most population-based studies have a follow-up of more than 3 years. 5 14 15 16 17

To the best of our knowledge, this is the first time that the presence of siblings is reported as a protective factor against a poor prognosis at 3 months and 1 year after FES. Since stroke rehabilitation at home could reduce disability, 23 we may speculate that the presence of sons and daughters could increase this form of rehabilitation and improve prognosis. Working before stroke was also a protective factor, probably associated with younger stroke subjects who have a better prognosis. 24 The presence of atrial fibrillation has been identified as a poor prognostic factor in a population-based study. 25 In our data, atrial fibrillation at admission tended to be a predictor of poor prognosis, although at a nonsignificant level. We found that heart failure was a predictor of poor prognosis at 1 year as in another study. 15 Preexisting dementia showed a nonsignificant tendency to a poor prognosis at 3 months but was significant at 1 year after a FES. The role of preexisting dementia in prognosis in ischemic stroke is controversial in the literature. 26 27

In our multivariable analysis, we found that, except for comorbidities, most predictors of poor prognosis after FES were not modifiable. These predictors may reflect underlying structural socioeconomic factors in a middle-income country, such as limited access to education and healthcare, which could not be confirmed by our study. Therefore, in addition to primary prevention strategies to prevent stroke, it is essential to improve access to better management during the acute phase. These are the main interventions aimed to reduce the impact of stroke prognosis in a higher-risk population. 28

## References

[1] Hardie K Hankey G J Jamrozik K Broadhurst R J Anderson C Ten-year survival after first-ever stroke in the perth community stroke study Stroke 2003 34 08 1842 184610.1161/01.STR.0000082382.42061.EE 12843343 10.1161/01.STR.0000082382.42061.EE

[2] Appelros P Nydevik I Viitanen M Poor outcome after first-ever stroke: predictors for death, dependency, and recurrent stroke within the first year Stroke 2003 34 01 122 12610.1161/01.str.0000047852.05842.3c 12511762 10.1161/01.str.0000047852.05842.3c

[3] Oxford Vascular Study Luengo-Fernandez R Paul N L Gray A M Population-based study of disability and institutionalization after transient ischemic attack and stroke: 10-year results of the Oxford Vascular Study Stroke 2013 44 10 2854 286110.1161/STROKEAHA.113.001584 23920019 10.1161/STROKEAHA.113.001584 PMC4946627

[4] Lavados P M Hoffmeister L Moraga A M Incidence, risk factors, prognosis, and health-related quality of life after stroke in a low-resource community in Chile (ÑANDU): a prospective population-based study Lancet Glob Health 2021 9 03 e340 e35110.1016/S2214-109X(20)30470-8 33422189 10.1016/S2214-109X(20)30470-8

[5] Cabral N L Nagel V Conforto A B Five-year survival, disability, and recurrence after first-ever stroke in a middle-income country: A population-based study in Joinvile, Brazil Int J Stroke 2018 13 07 725 73310.1177/1747493018763906 29513098 10.1177/1747493018763906

[6] Minelli C Cabral N L Ujikawa L T Trends in the Incidence and Mortality of Stroke in Matão, Brazil: The Matão Preventing Stroke (MAPS) Study Neuroepidemiology 2020 54 01 75 8210.1159/000503005 31586994 10.1159/000503005

[7] Instituto Brasileiro de Geografia e Estatística (IBGE) . (Accessed June 23, 2023). Available from:https://ww2.ibge.gov.br/home/estatistica/populacao/contagem/conceitos.shtm

[8] Macinko J Harris M J Brazil's family health strategy--delivering community-based primary care in a universal health system N Engl J Med 2015 372 23 2177 2181 26039598 10.1056/NEJMp1501140

[9] Aho K Harmsen P Hatano S Marquardsen J Smirnov V E Strasser T Cerebrovascular disease in the community: results of a WHO collaborative study Bull World Health Organ 1980 58 01 113 130 6966542 PMC2395897

[10] Cincura C Pontes-Neto O M Neville I S Validation of the National Institutes of Health Stroke Scale, modified Rankin Scale and BarthelIndex in Brazil: the role of cultural adaptation and structured interviewing Cerebrovasc Dis 2009 27 02 119 122 19039215 10.1159/000177918 PMC7065394

[11] Bamford J Sandercock P Dennis M Burn J Warlow C Classification and natural history of clinically identifiable subtypes of cerebral infarction Lancet 1991 337 8756 1521 1526 1675378 10.1016/0140-6736(91)93206-o

[12] International Stroke Incidence Collaboration Sudlow C LM Warlow C P Comparable studies of the incidence of stroke and its pathological types: results from an international collaboration Stroke 1997 28 03 491 499 9056601 10.1161/01.str.28.3.491

[13] Austin P C White I R Lee D S van Buuren S Missing Data in Clinical Research: A Tutorial on Multiple Imputation Can J Cardiol 2021 37 09 1322 133110.1016/j.cjca.2020.11.010 33276049 10.1016/j.cjca.2020.11.010 PMC8499698

[14] Hankey G J Jamrozik K Broadhurst R J Forbes S Anderson C S Long-term disability after first-ever stroke and related prognostic factors in the Perth Community Stroke Study, 1989-1990 Stroke 2002 33 04 1034 104010.1161/01.str.0000012515.66889.24 11935057 10.1161/01.str.0000012515.66889.24

[15] ASTRO study group Feigin V L Barker-Collo S Parag V Auckland Stroke Outcomes Study. Part 1: Gender, stroke types, ethnicity, and functional outcomes 5 years poststroke Neurology 2010 75 18 1597 160710.1212/WNL.0b013e3181fb44b3 21041783 10.1212/WNL.0b013e3181fb44b3

[16] Aked J Delavaran H Lindgren A G Survival, causes of death and recurrence up to 3 years after stroke: A population-based study Eur J Neurol 2021 28 12 4060 406810.1111/ene.15041 34327786 10.1111/ene.15041

[17] Kiyohara Y Kubo M Kato I Ten-year prognosis of stroke and risk factors for death in a Japanese community: the Hisayama study Stroke 2003 34 10 2343 234710.1161/01.STR.0000091845.14833.43 14500930 10.1161/01.STR.0000091845.14833.43

[18] Goulart A C Bensenor I M Fernandes T G Alencar A P Fedeli L M Lotufo P A Early and one-year stroke case fatality in Sao Paulo, Brazil: applying the World Health Organization's stroke STEPS J Stroke Cerebrovasc Dis 2012 21 08 832 83810.1016/j.jstrokecerebrovasdis.2011.04.017 21705233 10.1016/j.jstrokecerebrovasdis.2011.04.017

[19] Castro H HG Alencar A P Benseñor I M Lotufo P A Goulart A C Multimorbidities Are Associated to Lower Survival in Ischaemic Stroke: Results from a Brazilian Stroke Cohort (EMMA Study) Cerebrovasc Dis 2017 44(3-4):232 23910.1159/000479827 28848194 10.1159/000479827

[20] de Carvalho J J Alves M B Viana GÁ Stroke epidemiology, patterns of management, and outcomes in Fortaleza, Brazil: a hospital-based multicenter prospective study Stroke 2011 42 12 3341 334610.1161/STROKEAHA.111.626523 22052521 10.1161/STROKEAHA.111.626523

[21] Cisse F A Ligot N Conde K Predictors of stroke favorable functional outcome in Guinea, results from the Conakry stroke registry Sci Rep 2022 12 01 112510.1038/s41598-022-05057-6 35064178 10.1038/s41598-022-05057-6 PMC8782910

[22] Sudlow C L Warlow C P Comparing stroke incidence worldwide: what makes studies comparable? Stroke 1996 27 03 550 55810.1161/01.str.27.3.550 8610328 10.1161/01.str.27.3.550

[23] Rasmussen R S Østergaard A Kjær P Stroke rehabilitation at home before and after discharge reduced disability and improved quality of life: a randomised controlled trial Clin Rehabil 2016 30 03 225 23610.1177/0269215515575165 25758941 10.1177/0269215515575165

[24] van Almenkerk S Smalbrugge M Depla M F Eefsting J A Hertogh C M What predicts a poor outcome in older stroke survivors? A systematic review of the literature Disabil Rehabil 2013 35 21 1774 178210.3109/09638288.2012.756941 23350761 10.3109/09638288.2012.756941

[25] Lin H J Wolf P A Kelly-Hayes M Stroke severity in atrial fibrillation. The Framingham Study Stroke 1996 27 10 1760 176410.1161/01.str.27.10.1760 8841325 10.1161/01.str.27.10.1760

[26] Saposnik G Kapral M K Cote R Is pre-existing dementia an independent predictor of outcome after stroke? A propensity score-matched analysis J Neurol 2012 259 11 2366 237510.1007/s00415-012-6508-4 22532173 10.1007/s00415-012-6508-4

[27] Zupanic E von Euler M Winblad B Mortality After Ischemic Stroke in Patients with Alzheimer's Disease Dementia and Other Dementia Disorders J Alzheimers Dis 2021 81 03 1253 126110.3233/JAD-201459 33935077 10.3233/JAD-201459 PMC8293632

[28] Rigual R Fuentes B Díez-Tejedor E Management of acute ischemic stroke Med Clin (Barc) 2023 161 11 485 49210.1016/j.medcli.2023.06.022 37532617 10.1016/j.medcli.2023.06.022

